# Molecular Mechanisms of Melatonin-Mediated Cell Protection and Signaling in Health and Disease

**DOI:** 10.3390/pharmaceutics13020129

**Published:** 2021-01-20

**Authors:** Dalia M. Kopustinskiene, Jurga Bernatoniene

**Affiliations:** 1 Institute of Pharmaceutical Technologies, Faculty of Pharmacy, Medical Academy, Lithuanian University of Health Sciences, Sukileliu pr. 13, LT-50161 Kaunas, Lithuania; DaliaMarija.Kopustinskiene@lsmuni.lt; 2 Department of Drug Technology and Social Pharmacy, Faculty of Pharmacy, Medical Academy, Lithuanian University of Health Sciences, Sukileliu pr. 13, LT-50161 Kaunas, Lithuania

**Keywords:** melatonin, antioxidant, cardiolipin, mitochondria, cardioprotection, neuroprotection, tumor suppression

## Abstract

Melatonin, an endogenously synthesized indolamine, is a powerful antioxidant exerting beneficial action in many pathological conditions. Melatonin protects from oxidative stress in ischemic/reperfusion injury, neurodegenerative diseases, and aging, decreases inflammation, modulates the immune system, inhibits proliferation, counteracts the Warburg effect, and promotes apoptosis in various cancer models. Melatonin stimulates antioxidant enzymes in the cells, protects mitochondrial membrane phospholipids, especially cardiolipin, from oxidation thus preserving integrity of the membranes, affects mitochondrial membrane potential, stimulates activity of respiratory chain enzymes, and decreases the opening of mitochondrial permeability transition pore and cytochrome *c* release. This review will focus on the molecular mechanisms of melatonin effects in the cells during normal and pathological conditions and possible melatonin clinical applications.

## 1. Introduction

The natural hormone melatonin (*N*-acetyl-5-methoxytryptamine) can be found in almost all species, such as fungi, plants, and animals [1]. The diet sources rich in melatonin include olives, rice, chamomile, green tea, coffee, tomatoes, and cereals [2,3]. Melatonin modulates many physiological functions, including sleep and circadian rhythm regulation, neuro- and cardioprotection, acting as powerful antioxidant and protecting from lipid peroxidation, inflammation, decreasing tumor growth, inducing apoptosis, and enhancing mitochondrial activity [1,4].

## 2. Melatonin Biosynthesis and Bioavailability

**Figure 1:**
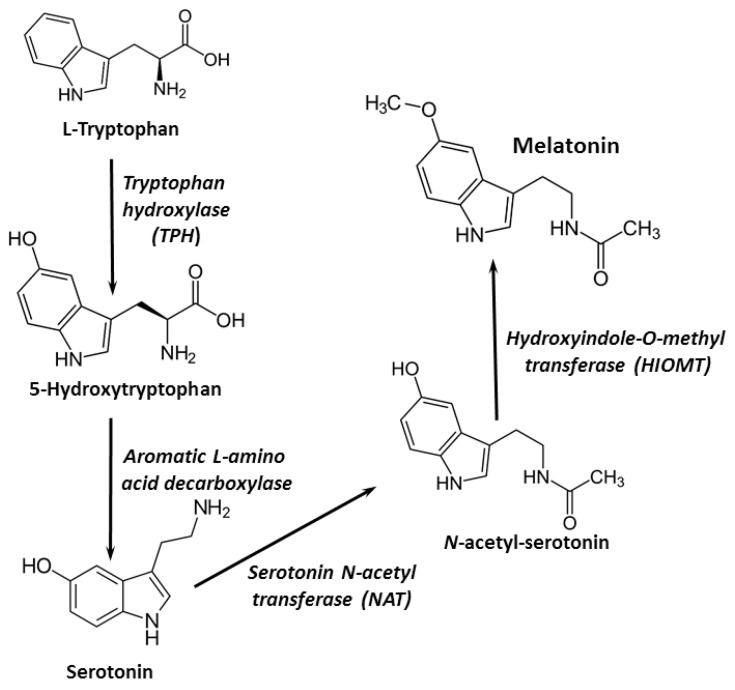
Melatonin synthesis pathways.

Melatonin is secreted primarily by the pineal gland, where it is synthesized from the amino acid tryptophan. Melatonin synthesis could also occur in other organs and cells, such as the bone marrow, brain, lens, skin, retina, and lymphocytes. Four essential enzymes are involved in melatonin synthesis: tryptophan-5-hydroxylase, 5-hydroxytryptophan decarboxylase, serotonin-*N*-acetyltransferase, and hydroxyindole-*O*-methyltransferase [5] (Figure 1). Around 30 µg/day of melatonin is secreted constantly in adult humans; however, in the evening, its synthesis increases reaching a maximum plasma peak in the middle of the dark period. As a neurohormone, melatonin is mainly involved in circadian rhythm control [1,6].

Melatonin is not retained in the pineal gland; it is secreted to the blood and transported to the liver, where it is rapidly metabolized [7]. First, cytochrome P450 monooxygenases A2 and 1A hydroxylate melatonin at the C6 position, then melatonin is converted to its sulfate derivative (6-sulfatoxymelatonin), and later excreted in the urine [7].

Melatonin is transported bound to the serum albumin in the blood, although it could be bound by hemoglobin as well [8,9]. It is capable of crossing the blood–brain barrier [10,11] and cellular membranes [12], and is found in high amounts in mitochondria within the cells [13,14].

The distribution half-life of melatonin is 0.5–5.6 min after intravenous administration [15,16]. After the administration of usual oral dosages (1–5 mg), the plasma concentration peak is reached after 60 min, and then rapidly decreases in a biphasic manner, with the corresponding half-life values of 2 and 20 min [15,16]. Basal melatonin concentrations are reached in 4–8 h. In healthy volunteers, an administration of 0.5 mg of melatonin resulted in a plasma melatonin peak varying between 2 and 395 nmol/L and an elimination half-life of 47 min, whereas bioavailability was around 33%, ranging from 10 to 56% [15,16]. The low melatonin bioavailability is due to excessive hepatic first pass metabolism [15,16,17,18].

The synthesis of melatonin decreases with age, often causing insomnia; the largest decline could be observed in the cases of Alzheimer’s disease, cardiovascular issues, and cancer [19]. Usually, melatonin is safe and not toxic; even at high doses only mild side effects, such as dizziness, headaches, nausea, and sleepiness, could be observed [20].

## 3. Receptor-Dependent Effects of Melatonin

**Figure 2:**
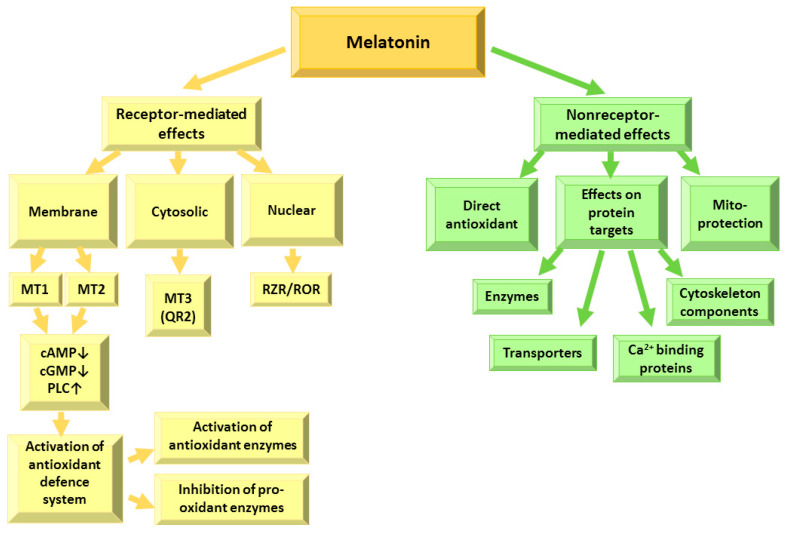
Receptor-dependent and receptor-independent effects of melatonin. MT—melatonin receptor; cAMP—adenylyl cyclase; cGMP—guanylyl cyclase; PLC—phospholipase C; QR2—quinone reductase 2; RZR/ROR—nuclear retinoid Z receptor.

Melatonin exerts its effects within the cells via interactions with receptors and with intracellular targets, such as transporters, ion binding proteins, enzymes, cytoskeletal components and mitochondria [9,14,21,22], due to its ability to cross freely membranes as well as penetrate the blood–brain barrier [12] (Figure 2).

Melatonin receptors are spread in the retina, brain, kidneys, gastrointestinal tract, skin, immune, endocrine, reproductive, and cardiovascular systems [5]. They modulate adenylyl cyclase (cAMP), guanylyl cyclase (cGMP) and phospholipase C (PLC) activities—and subsequently, calcium and potassium fluxes in the cell [23,24]. Melatonin receptors 1 (MT1) and 2 (MT2) are composed of 350 amino acids and 362 amino acids, respectively, and are 60% homologous proteins belonging to the class of G-protein-coupled receptors (GPCR) [23] with four intracellular and four extracellular domains and 7-transmembrane-helices [25]. The MT1 receptor has an approximately three-fold higher affinity to melatonin than the MT2 receptor, and is thought to be responsible for the circadian effects of melatonin and modulation of the signal transduction in the reproductive system and regulation of peripheral vasoconstriction [26,27]. MT1 receptors are expressed in the retina, brain, cardiovascular system, immune system, skin, pancreas, kidney, liver, spleen, adrenal cortex, testes, ovary, placenta, and breast [26,27]. Most MT2 receptors are present in the retina, brain, immune system, adipose tissue, blood vessels, mammary glands, testes, kidney, and gastrointestinal tract [28]. Melatonin binding to MT1 and MT2 receptors results in the inhibition of the AC/cAMP/PKA/CREB signaling pathway [29], leading to the activation of calcium signaling by calmodulin kinases and PKC and regulation of hormone synthesis. Melatonin binding to the receptors could also activate the Rafs/MEK1/2/ERK1/2 signaling pathway [30,31], which is important for cell proliferation regulation [28]. The melatonin-modulated ERK–MAPK/JNK signaling pathway plays a major role in the oxidative stress [32]. Melatonin can activate the PI3K/Akt (PKB) signaling pathway [33,34] which is very important in cardioprotection [33]. Furthermore, melatonin can decrease tumor cell proliferation and viability by inhibiting the negative feedback from the downstream effector of the PI3K/Akt (PKB) signaling pathway—mammalian target of rapamycin complex 1 (mTOR) [35,36] and modulate apoptosis [37,38]—and inhibit the GC/cGMP/PKG pathway [39]. Melatonin receptor 3 (MT3) is found in animals and is a quinone reductase 2 (QR2) capable of neutralizing free radicals [40,41,42]. The melatonin effects on the nuclear retinoid Z receptor (ROR/RZR) protein family [9,43] play a role in melatonin immunomodulatory actions—T and B lymphocyte stimulation, inhibition of cytokine production, and suppression of nF-κB-dependent inflammation [43]. Thus, melatonin regulates many physiological functions via receptor-dependent signaling pathways.

**Figure 3:**
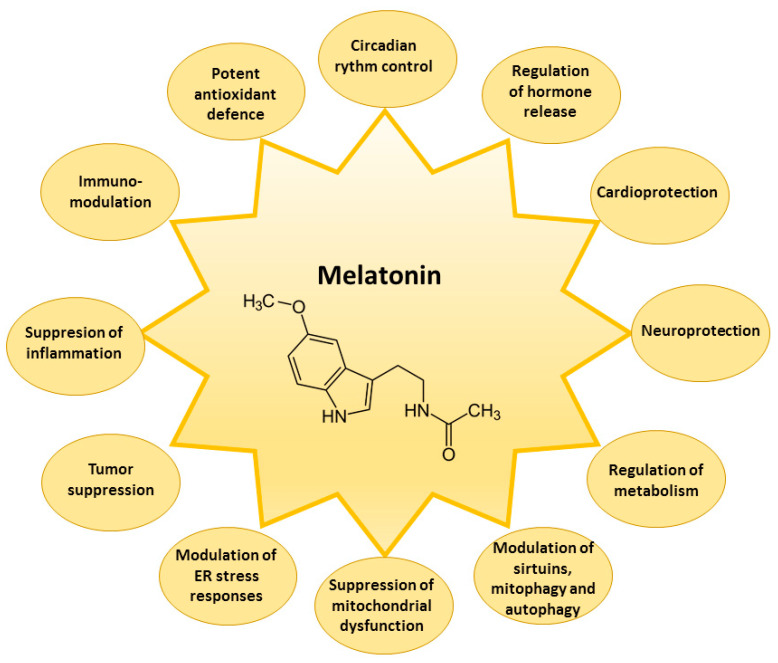
Main biological effects of melatonin in the cell. ER—endoplasmic reticulum.

The receptor-related melatonin effects comprise the indirect modulation of the activity of the enzymes involved in cellular protection against damage by reactive oxygen species (ROS) and reactive nitrogen species (RNS) [7,44,45]. Reduced glutathione (GSH) is one of the most important components of the first line of the antioxidant defense system in the cells capable of neutralizing ROS formed in a variety of redox reactions. The activities of enzymes that improve intracellular levels of reduced GSH are maintained by melatonin [46]. Melatonin increases the activity of γ-glutamylcysteine synthase, which is the rate-limiting enzyme of the GSH synthesis pathway, thus maintaining the cellular levels of GSH [47,48]. Furthermore, melatonin can regulate GSH/GSSG (oxidized glutathione) balance by the stimulation of glutathione reductase (GR), responsible for GSSG reduction to GSH [46]. Melatonin can enhance the gene expression of antioxidant enzymes, comprising superoxide dismutase (SOD) and glutathione peroxidase (GSH-Px) [49,50], and increase catalase (CAT) activity [19]. Melatonin as an indirect antioxidant can also downregulate pro-oxidative and pro-inflammatory enzymes [51]. Melatonin suppresses the nitric oxide synthase, responsible for nitric oxide, and lipoxygenase, for superoxide anion generation [52]. Downregulation of lipoxygenase activity by melatonin protects the cells from the hydroperoxidation of polyunsaturated fatty acids [53]. Melatonin modulates endoplasmic reticulum stress responses [54], activity of sirtuins [55], and processes of mitophagy and autophagy [56,57]. Thus, melatonin is a powerful, multifaceted, natural antioxidant capable of preventing the overproduction of ROS and RNS, to protect the functions of many biological molecules—DNA, lipids, and proteins—from oxidative damage, and to suppress the development of severe degenerative disorders such as cardiologic and neurological diseases, diabetes, and cancer [49,50] (Figure 3).

## 4. Receptor-Independent Effects of Melatonin

### 4.1. Direct Antioxidant Effects of Melatonin

Melatonin is a potent direct scavenger of free radicals, including singlet oxygen, hydroxyl, peroxyl radicals, hydrogen peroxide, nitric oxide and peroxynitrite. Melatonin effectively terminates the radical reaction chain without the formation of pro-oxidant metabolites [3,58] by donating one or more electrons to the free radicals [59]. It is very important that melatonin intermediates produced during free radical neutralization reactions—cyclic 3-hydroxymelatonin, *N*1-acetyl-*N*2-formyl-5-methoxykynuramine and *N*-acetyl-5-methoxykynuramine—are also strong antioxidants [19]; thus, one melatonin molecule has the ability to scavenge up to 10 ROS compared to the traditional antioxidants that typically neutralize one ROS [19,51]. Melatonin can react with hydroxyl radical resulting in the formation of the indolyl radical cation, which has low reactivity and toxicity [60]. Melatonin also directly scavenges the alkoxyl radical, produced during the transition metal-catalyzed degradation of lipid peroxides, thus preventing further lipid peroxidation [61,62]. Melatonin also can act as metal chelator capable of reducing metal-induced toxicity [63].

### 4.2. Effects of Melatonin on Target Proteins

Melatonin can directly interact with various proteins within the cell, including enzymes, transporters, Ca^2+^-binding proteins, and cytoskeleton components [9].

The zinc-dependent matrix metalloproteinases (MMPs) which are related to extracellular matrix remodeling and important in pathological processes such as tumor proliferation and the formation of metastasis, atherosclerosis, rheumatoid arthritis, and gastric ulcers, have been suggested to be one of the targets of melatonin [64]. Potent anti-ulcer action of melatonin is attributed to its possible binding to pepsin—a stomach protease responsible for breaking down the ingested proteins into peptides [65,66]. Melatonin-induced inhibition of phosphoprotein phosphatase 2A protects from the hyperphosphorylation of neuronal proteins, protecting neuronal cells and alleviating neurodegenerative diseases [67].

Melatonin is highly lipophilic and can therefore be distributed in the body by the means of passive diffusion [68]. Besides this mechanism, melatonin can be transported across the plasma membrane by the glucose transporter GLUT1 [37,69] and across the mitochondrial membranes by the oligopeptide transporters PEPT1/2 [70]. The melatonin binding site on GLUT1 overlaps with glucose binding [69], which might play a role in the counteraction of the Warburg effect in cancer cells by melatonin [71]. In the plasma, melatonin is bound by serum albumin [8,9].

Melatonin is a high affinity ligand of a Ca^2+^-binding protein calmodulin, participating in many calcium signaling pathways [28,72]. The cytoskeletal effects of melatonin were suggested to be modulated by the Ca^2+^–calmodulin complex, although at higher concentrations melatonin has been observed to bind tubulin [73,74,75], leading to the inhibition of microtubule formation. Melatonin via interactions with cytosolic protein networks can control cell motility, division, and organelle function, as well as in mitogen-activated protein kinase (MAPK)-related scaffold protein trafficking and signaling functions [28]. Melatonin can inhibit cytoskeleton reorganization; thus, suppressing cancer proliferation and restoring normal mitochondrial functions [76,77,78]. Melatonin has been shown to bind calreticulin—a Ca^2+^-binding protein known for its chaperon action and responsible for the regulation of Ca^2+^ homeostasis [79]. More than 15 melatonin target proteins have been proposed, capable of binding melatonin at concentrations ranging from subnanomolar to millimolar, and indicating the wide variety of processes that could be modulated by melatonin in the cells [9].

### 4.3. Effects of Melatonin on Epigenetic Regulation

MicroRNAs (miRNAs) have emerged as modulators of gene expression, capable of reaching various parts of organisms via their distribution in exosomes and ectosomes [80], and playing important roles in the development of many pathological conditions [80]. Melatonin has been shown to exert the protective activity by inducing changes in miRNA expression [80,81,82,83].

In a murine model, melatonin reduced the impairment of alcoholic liver disease by enhancing miRNA-497 expression [84], alleviated liver steatosis by modulating miRNA-23a expression [85], and reduced primary sclerosing cholangitis in a murine model of liver fibrosis by inhibiting miRNA-200b expression in cholangiocytes and stellate cells [86].

In Alzheimer’s disease models, melatonin partially suppressed neuroinflammation by reducing an increase in miRNA-124 and enhancing miRNA-132 [87]. Melatonin alleviated neonatal brain inflammation in rats induced by bacterial lipopolysaccharide by restoring upregulated miRNA-34a and downregulated miRNA-146a and miR-126 activities [88].

In cancer models, melatonin suppressed miRNA-155 expression in several human glioma cell lines [89], inhibited angiogenesis modulated by miRNA-3195 and miRNA-374b expression in hypoxic PC-3 prostate cancer cells [90], inhibited the expression of 10 miRNA, and enhanced the expression of 12 miRNA in MCF-7 breast cancer cells [91]. Future directions for studies of the miRNA and melatonin interactions would be the clarification of whether melatonin could modulate the changes in the composition of exosomal and ectosomal miRNAs, thus affecting the progression of pathological conditions in the organism [80].

### 4.4. Effects of Melatonin on Mitochondrial Functions

**Figure 4:**
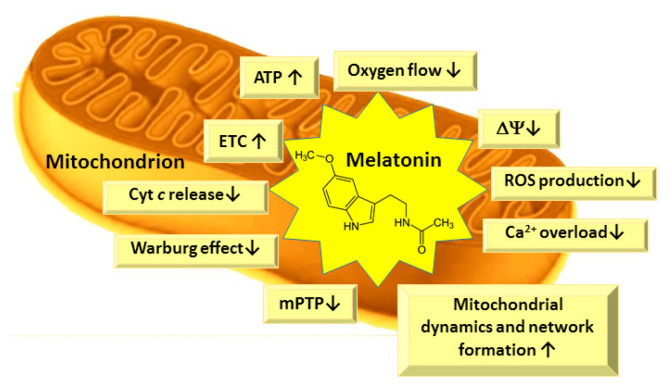
Main biological effects of melatonin in mitochondria. ETC—electron transport chain; ΔΨ—mitochondrial membrane potential; ROS—reactive oxygen species; mPTP—mitochondrial permeability transition pore; Cyt—cytochrome.

Several studies indicate that, in subcellular compartments, melatonin may be differentially distributed. Cell nuclei and mitochondria, in particular, tend to contain higher levels of melatonin than other compartments, such as cytosol [70]. Mitochondria play a crucial role in metabolism, calcium homeostasis, apoptosis, and regulation of many physiological and pathological processes in the cells [92]. Mitochondria are essential organelles, responsible for cellular energy supply through oxidative phosphorylation as well as being the main site of ROS generation. ROS-induced impairment of mitochondrial and cellular functions can be controlled by either preventing the ROS formation or by scavenging them as soon as they are produced [93]. Mitochondria are the main sites for melatonin synthesis and metabolism [70,94], thus melatonin could be an ideal mitochondrial protector due to its close location to ROS production sites and its potent antioxidant properties (Figure 4).

Melatonin has been shown to improve mitochondrial functions, to stimulate the activity of the respiratory chain—mainly complexes I, III and IV [4,95,96,97,98,99]—and to increase mitochondrial ATP production in both normal and pathological conditions, although it did not change the activity of the ATP synthase [4,95,96,97,98,99]. The maintained ATP production at decreased oxygen flux and lower mitochondrial membrane potential is similar to the mild uncoupling, which is important for cardioprotection, neuroprotection, and the prevention of various pathological conditions due to decreased ROS generation [100,101]. Furthermore, the increased ATP production protects from the collapse of mitochondrial membrane potential and calcium overload and inhibits opening of the mitochondrial permeability transition pore (mPTP)—and subsequently, the cell death [102,103]. Peroxidation of mitochondrial membrane phospholipids leads to mitochondrial dysfunction in aging and is the main cause of age-associated disorders [97,104,105,106]. The structure of membrane phospholipids is altered during peroxidation, resulting in the structural changes of the lipid bilayer, and altered membrane fluidity and permeability. Changed membrane properties influence the impairments in the mitochondrial electron transport and oxidative phosphorylation, inner membrane impermeability, decreased mitochondrial membrane potential and effects on mitochondrial Ca^2+^ homeostasis [107,108,109]. There is a high content of unsaturated fatty acids, such as linoleic acid, in cardiolipin molecules in the heart and liver, and arachidonic and docosahexaenoic acids in the cardiolipin molecules of brain tissue mitochondria, which are highly sensitive to ROS-induced damage [104,110]. Cardiolipin is present in the inner mitochondrial membrane, especially in intermembrane contact sites, near to the sites of ROS production, i.e., the respiratory chain complexes I and III [104,110]. The oxidation or decrease in cardiolipin in mitochondrial inner membrane is supposed to be responsible for age-related decline in mitochondrial functions [102,104]. Oxidized cardiolipin does not tightly bind cytochrome *c* (Cyt *c*) and it is released into the intermembrane space, then due to alterations in lipid bilayer, mPTP is formed [111], and Cyt *c* can be released to cytosol as a signal molecule for apoptosis processes. Melatonin has been shown to prevent cardiolipin peroxidation in mitochondria both under in vitro and in vivo conditions [102,106,112], thus preventing mPTP opening and cytochrome *c* release from mitochondria [113].

Melatonin also maintains mitochondrial bioenergetics and redox homeostasis via the regulation of mitochondrial dynamics [114]. Studies have revealed the importance of mitochondrial dynamics in inflammatory responses and the maintenance of immune synapse stability. Mitochondrial dynamics have been implicated to modulate the antigen-specific activation and immune responses [115]. Mitochondria have been shown to move towards the immune synapse, driven by cell polarization and integrin adhesion [115,116,117], as well as the fission factor dynamin-related protein 1 (Drp1) regulating mitochondrial redistribution in response to T cell receptor assembly [116].

Melatonin has been shown to exert multiple anticancer effects related to mitochondrial function regulation. A fundamental protective mechanism for reducing pathologies may be the ability of melatonin to alter metabolism in cancer cells as well as other metabolically compromised cells due to the suppression of aerobic glycolysis and activation of oxidative phosphorylation [71,118,119]. Besides inhibiting aerobic glycolysis, melatonin also down-regulates survival signaling and metastasis formation [38]. Melatonin modulates the activity of pyruvate kinase complex and suppresses the pyruvate dehydrogenase kinase, which is considered to be a potential therapeutic target in cancer [120]. The restoration of the mitochondrial network by melatonin increases apoptosis, thus reducing cell growth in lung, breast, and colon cancers [121,122,123].

Thus, melatonin, via its direct ROS/RNS scavenging at its production site—mitochondria—indirect activation of antioxidant defense system, and preservation of the integrity of mitochondrial membranes by protecting them from cardiolipin loss and its peroxidation, has a crucial role in maintaining normal physiological functions of mitochondria and energy turnover in the cells. Melatonin is highly present in mitochondrial membranes; therefore, future studies of the effects of melatonin on the functions of mitochondria in various tissues and organs under normal and pathological conditions as well as investigations of mitochondria-targeted melatonin preparations could help to elucidate the detailed mechanisms of the protective actions of melatonin and its role in alleviating many pathological conditions.

## 5. Beneficial Effects of Melatonin in Pathological Conditions

### 5.1. Effects of Melatonin in Neurodegenerative Disoders

Age-related conditions that share mitochondrial dysfunction, oxidative/nitrosative stress, and apoptosis in multiple parts of the brain are neurodegenerative disorders such as Alzheimer’s disease and Parkinson’s disease [124,125]. Reduced melatonin levels are found in the blood and cerebrospinal fluid of Alzheimer’s patients, even in the early onset of the disease [126]. In a rat model of sporadic Alzheimer’s disease, melatonin attenuated memory decline, amyloid-β accumulation, and neurodegeneration [127]. In a Parkinson’s disease model, melatonin restored electron transport chain complex I and complex IV activities, and downregulated mitochondrial inducible nitric oxide synthase by decreasing nitric oxide radicals in mitochondria from substantia nigra and striatum [128]. Decreased local melatonin synthesis in neuronal and immune cells, as well as in the glia and gut, might be important to the etiology and management of Parkinson’s disease [129]. Melatonin has been shown to be neuroprotective in various models of neurodegenerative diseases (for reviews see refs [56,57,130,131,132]). Clinical trials have also been performed to investigate the role of melatonin supplementation on the alleviation of clinical symptoms during Alzheimer’s disease [130]. In studies evaluating sleep efficiency, contradictory results were obtained—melatonin did not show beneficial effects in several studies [133,134,135], probably due to the stability of the assessment method used [130]. A slight neuroprotective effect of melatonin was observed when subjective criteria (Pittsburg Sleep Quality Index) [136] or actigraphy [137] were used to evaluate the melatonin effects. Nevertheless, the majority of clinical investigations support the beneficial effects of melatonin on cognitive impairment and sleep disorders [130].

### 5.2. Cardioprotective Effects of Melatonin

Melatonin is a cardioprotective agent, reducing the cardiac damage and changes in cellular physiology during ischemia/reperfusion injury due to actions at mitochondrial level [99,112,138,139]. Melatonin treatment of an ischemic/reperfused rat heart significantly decreased the level of lipid peroxidation, restored decreased mitochondrial respiration rate in State 3, as well as complex 1 and complex III activities in isolated rat heart mitochondria [22,112]. Furthermore, melatonin treatment resulted in a decrease in H_2_O_2_ generation, protected from cardiolipin loss, and its peroxidation thus preserving the physiological functions of mitochondrial membranes, the ETC activity, and preventing mPTP opening and cytochrome *c* loss [112,113,139,140]. Melatonin pretreatment attenuates IR-induced mitochondrial oxidative damage via the activation of the JAK2/STAT3 signaling pathway [141]. The STAT3 is a transcription factor of the manganese superoxide dismutase gene, which can increase manganese superoxide dismutase antioxidant activity, thus improving mitochondrial antioxidant defense [142]. Moreover, melatonin can also up-regulate mitochondrial STAT3 through the SAFE signal transduction pathway to reduce myocardial ischemia/reperfusion injury [143]. During ischemia/reperfusion in diabetic rats, melatonin protected mitochondrial functions by alleviating mitochondrial oxidative stress and stimulating mitochondrial biogenesis via up-regulation of the AMPK–PGC-1α–silent information regulator 3 (SIRT3) axis [144].

### 5.3. Effects of Melatonin in Diabetes, Obesity and Metabolic Diseases

Metabolic diseases—cardiovascular diseases, diabetes, obesity, and metabolic syndromes—are caused by the altered normal physiological processes in the organism [145,146,147]. Melatonin receptors MT1 and MT2 are expressed in islets of Langerhans and they can modulate α-cell-dependent glucagon secretion and β-cell-dependent insulin secretion [148]. Altered signaling of melatonin receptors, especially MT2 activation, could lead to the development of type-2 diabetes mellitus [148]. Melatonin concentration changes are important for the blood glucose-regulating function of the islets [145,148]. The inverse relationship between insulin and melatonin secretion was reported in the studies on diabetic rat models [148]. Furthermore, melatonin was able to inhibit glucose-induced insulin release in rodents [145,149]. Elevated insulin and leptin levels could suppress the increase in melatonin synthesis at night [150]. Additionally, the aging-related increase in insulin resistance could be prevented by melatonin as well [150]. Melatonin modulated body weight regulation and restored the insulin sensitivity in obese young rats on a high-fat diet [151,152]. Melatonin also decreased abdominal fat and plasma leptin levels in middle-aged rats [153,154]. Thus, melatonin could prevent body weight gain without affecting the food intake [145,154,155,156], due to elevated energy expenditure, mainly in brown adipose tissue [156,157] or a stimulation of physical activity or basal metabolic rate [145]. Hypo-lipidemic effects of melatonin were reported in rodents [152,158] and humans [159,160]. In peri- and post-menopausal women, melatonin up-regulated their high-density-lipoprotein cholesterol levels, but not the concentrations of total cholesterol [161]. Melatonin also decreased low-density-lipoprotein cholesterol and triglyceride levels simultaneously, increasing high-density-lipoprotein cholesterol levels in diabetic patients [159,160,162]. Further investigations of melatonin’s influence on mitochondrial functions in obesity or diabetes are expected to detail its mechanisms of alleviation of metabolic diseases [145,146,147].

### 5.4. Effects of Melatonin in Skeletal Muscle Disorders

The decreased level of melatonin in postmenopausal women was reported in sarcopenia [163,164]. Melatonin supplementation protected mitochondria from aging-caused impairment in cardiac and diaphragm muscles in the model of accelerated aging in mice [165]. Melatonin reduced the inflammation and increased glycolytic potential in skeletal muscles during sarcopenia [166]. Furthermore, melatonin protected from the development of sarcopenia in aged rodent models [167,168]. Melatonin protected from ischemic damage in rat cremaster and gracilis muscles during ischemia/reperfusion [169,170], and enhanced cell regeneration in rat muscle injury models [171,172]. In rodent fibromyalgia models, melatonin protected from ROS damage and restored mitochondrial dynamics parameters in the gastrocnemius muscle [164,173,174]. Melatonin, due to its antioxidant activity, was also effective in alleviating Duchenne muscular dystrophy in rodents and humans [175,176].

### 5.5. Immunomodulatory Effects of Melatonin

Melatonin exerts a potent immunomodulatory activity in vivo and in vitro [177,178,179]. Melatonin membrane receptors are present in T and B lymphocytes [69]. Melatonin promotes T cell differentiation from the type 1 helper T cells and regulates cytokine gene expression [180,181]. Furthermore, melatonin can enhance cytokine production in human peripheral blood mononuclear cells via its action on nuclear receptors [182].

Melatonin is a pleiotropic immunomodulatory agent, activating inflammation at an early phase via up-regulation of pro-inflammatory phosholipase A2, lipoxygenase, and cytokines (IL-1 and tumor necrosis factor alpha (TNF-α)) [183,184,185]. The anti-inflammatory action of melatonin is related to the suppression of Th1 function and activation of type 2 helper T lymphocytes which produce interleukin (IL)-4 [69].

Melatonin decreases NF-kB binding to DNA, probably by preventing its translocation to the nucleus, thus down-regulating cytokine production [186,187]. Melatonin suppresses chronic inflammation, inhibiting the expression of inducible nitric oxide synthase (iNOS), cyclooxygenase, as well as protein lipase A2, lipoxygenase and cytokine activities due to its antioxidant properties [188]. This activity is similar to that of flavonoids, acting as antioxidants under physiological and as pro-oxidants under pathological conditions.

### 5.6. Effects of Melatonin in Gastrointestinal Tract

Alterations in gut microbiota and increased gut permeability are implicated in the etiology, course, and treatment of many neurodegenerative diseases [129]. Moreover, the exposure to stress induces changes in the brain–gut interactions, leading to the development of gastrointestinal disorders [189]. Melatonin is present in significant amounts in the gastrointestinal tract, directly affecting its tissues and indirectly modulating the brain–gut axis [129,190,191]. Melatonin protects from the increase in the ROS-induced lipid peroxidation and up-regulates key anti-oxidizing enzymes such as superoxide dismutase [129]. Melatonin restores gastric and pancreatic functions and is implicated in the healing of gastric ulcers, thus beneficially affecting the brain–gut axis [190,191,192,193].

### 5.7. The Role of Melatonin in the Treatment of the Protozoan Parasitic Infections

Melatonin has been shown to be beneficial in bacterial, viral, and parasitic infections due to its potent antioxidant immunomodulatory effects and direct effects on mitochondrial functions [194]. Melatonin suppressed the development of Plasmodium parasites in malaria due to regulation of cAMP–PKA and IP3–Ca^2+^ signaling pathways [195,196,197,198]. In Chagas’ disease [199], toxoplasmosis [200] and African trypanosomiasis [201], melatonin stimulated the host’s immune response against the parasite, up-regulating the production of inflammatory mediators. Melatonin inhibited leishmaniasis parasite replication via direct effects on mitochondrial functions—activating the efflux of Ca^2+^ from mitochondria, increasing the level of mitochondrial nitrites, and suppressing the superoxide dismutase activity [202]. Serum melatonin levels were elevated in giardiasis patients, implicating that the increased melatonin concentrations could modulated phagocytic activity [203]. In amoebiasis, melatonin decreased the amoebic lesions and enhanced the leukophagocytosis [204].

Melatonin, such as many other antiparasitic drugs—ivermectin [205,206,207,208,209], doxycycline [210,211,212], metronidazole [213], mefloquine [214], and hydroxychloroquine [215,216]—has been suggested as a possible adjuvant agent in the treatment of coronavirus disease 2019 (COVID-19) infection [217,218,219,220,221]. Due to its anti-inflammatory, antioxidant and potent mitoprotective properties, melatonin might be beneficial in the preservation of mitochondrial functions and neuro- and cardioprotection during infections, whereas clarification of its mechanism of action as antiparasitic agent would be an interesting topic for further research.

## 6. Conclusions and Future Perspectives

Melatonin, an endogenous hormone synthetized from tryptophan in the pineal gland mainly during the dark period of the day, regulates the circadian rhythm, can cross cellular membranes and the blood–brain barrier, and is finally driven to mitochondria, where it protects from ROS directly at their generation site—the mitochondrial electron transport chain. Melatonin is most powerful natural antioxidant, alleviating cellular injury during many pathological conditions, such as ischemia/reperfusion, neurodegenerative diseases, aging, and exerting anti-proliferative, anti-inflammatory, and anti-cancer effects.

Melatonin acts via receptor-dependent and independent pathways, modulating various signal transduction steps and supporting the healthy state of mitochondria, preventing the cellular energy supply from impairment. Melatonin is well tolerated as a supplement even at high doses (1–5 mg, [15,16,17,18]), and could serve as a potential adjuvant remedy in many degenerative and infectious diseases and for the sleep regulation. The novel pharmacological melatonin formulations such as prolonged-release tablets or melatonin liposomes, niosomes, or nanoparticles for alternative administration routes—e.g., intranasal or transdermal delivery—could be developed to overcome the problems related to the short therapeutic window of melatonin caused by the poor oral absorption and low bioavailability due to the high first-pass metabolism.

## Data Availability

Not applicable.
